# Supplementary material for: ACAT1/SOAT1 Blockade Suppresses LPS-Mediated Neuroinflammation by Modulating the Fate of Toll-like Receptor 4 in Microglia
Source: Int J Mol Sci. 2023 Mar 15;24(6):5616. doi: 10.3390/ijms24065616 (PMC10053317; doi:10.3390/ijms24065616)
Supplement: Supplementary file 1 [file ijms-24-05616-s001.zip › ijms-2244702-supplementary.pdf]

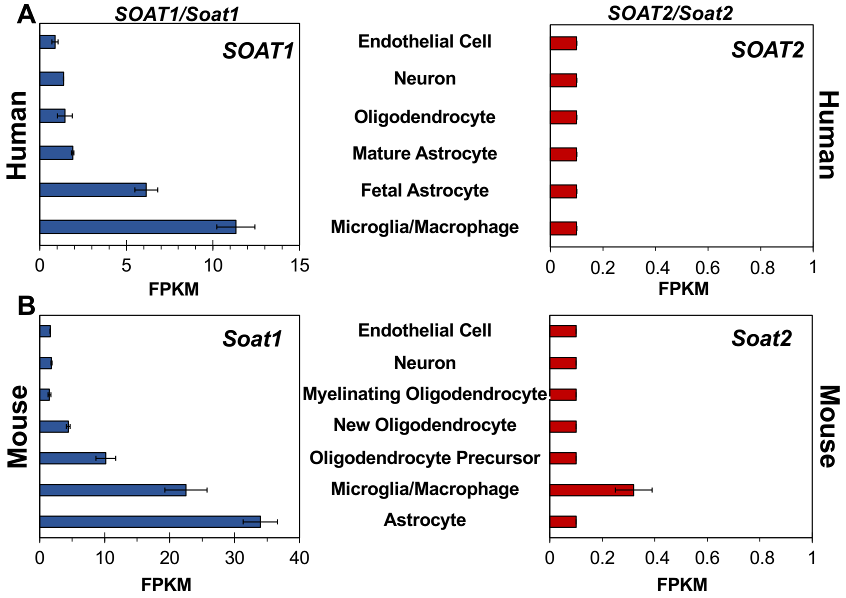

Figure S1. ACAT1/SOAT1, but not ACAT2/SOAT2, is significantly expressed in both human and mouse microglia. *(A) Microglia expresses high levels of ACAT1/SOAT1 in human brains and (B) Acat1/Soat1 expresses more in astrocytes in the mouse brains. Gene expression data are in fragments per kilobase million (FPKM). Data are retrieved from the Barres Laboratory website* ***[brainrnaseq.org]****. Error bars represent +/- 1 SEM.*

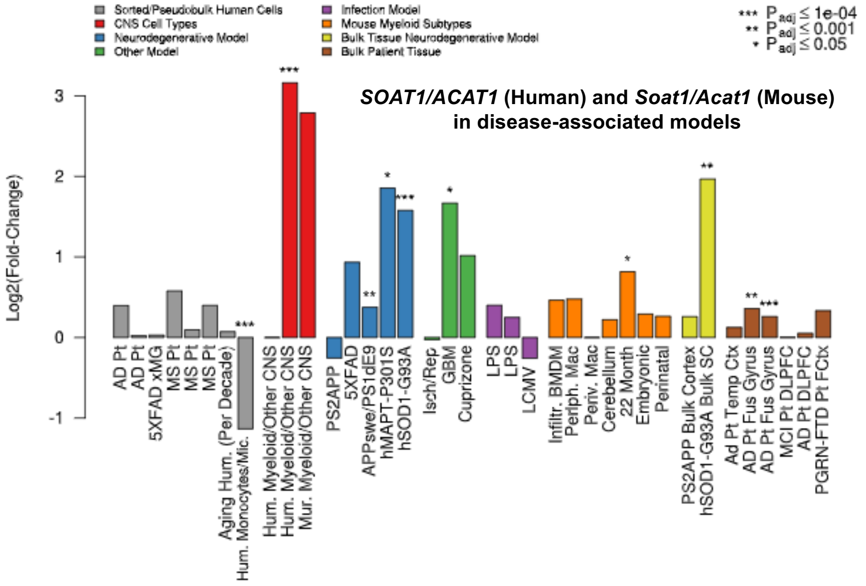

Figure S2. Acat1/Soat1 expression is up-regulated in several human patient brains and mouse models of neurological diseases. *Mouse model expression includes hMAPT(P301S), hSOD1-G93A, APP(Swedish)/PS1(dE9), hSOD1-G93A and Glioblastoma Multiforme. And in human AD patient tissue includes fusiform gyrus. Gene expression data are log2(fold-change). Data are retrieved from (***30)***.* *Error bars represent +/- 1 SEM.*
